# Mapping cumulative pressures on the grazing lands of northern Fennoscandia

**DOI:** 10.1038/s41598-022-20095-w

**Published:** 2022-09-30

**Authors:** Marianne Stoessel, Jon Moen, Regina Lindborg

**Affiliations:** 1 Department of Physical Geography, Stockholm University, 106 91 Stockholm, Sweden; 2 The Bolin Centre for Climate Research, Stockholm University, 106 91 Stockholm, Sweden; 3 Department of Ecology and Environmental Science, Umeå University, 901 87 Umeå, Sweden

**Keywords:** Climate-change impacts, Environmental impact, Sustainability, Environmental impact

## Abstract

Traditional grazing areas in Europe have declined substantially over the last century. Specifically, in northern Fennoscandia, the grazing land is disturbed by cumulative land-use pressures. Here we analysed the configuration of the grazing land for reindeer and sheep in northern Fennoscandia in relation to the concurrent land-use pressures from tourism, road and railway networks, forestry, industrial and wind energy facilities, together with predator presence and climate change. Our results show that 85% of the region is affected by at least one land-use pressure and 60% is affected by multiple land-use pressures, co-occurring with predator presence and rising temperatures. As such, a majority of the grazing land is exposed to cumulative pressures in northern Fennoscandia. We stress that, if the expansion of cumulative pressures leads to grazing abandonment of disturbed areas and grazing intensification in other areas, it could irreversibly change northern vegetation and the Fennoscandian mountain landscape.

## Introduction

In northern latitudes, extensive livestock grazing has a long history, where the use of semi-domesticated and domesticated grazers allowed human populations to settle and persist for thousands of years, mostly with sheep farming and reindeer herding^1,2^. In Fennoscandia (Norway, Sweden and Finland), about 40% of the land is assigned as reindeer grazing land^3,4^. In Sweden and Norway, reindeer husbandry and grazing are seen as a means of preserving the mountain landscape in its current state and is formulated as a national environmental goal^5,6^. Yet, extensive grazing is increasingly under cumulative pressures due to competing land-use activities that affect reindeer behaviour and herding practices, therefore changing the grazing patterns^2,7^. In addition, northern ecosystems are facing drastic shifts due to climate change^8^, which is predicted to result in increased productivity and climate-induced vegetation shifts that negatively affect northern specialist plant species^9–11^. Such climate-driven changes may interact with land-use changes in ways that can be either be additive, synergistic, or antagonistic^12^, ultimately affecting vegetation patterns and biodiversity.

During the last century, extensive livestock grazing has been replaced by intensive agriculture or forestry in Europe^13^. Intensification of human activities and land-use changes have been the main drivers of the decline of traditional grazing areas, resulting in a loss of 75% of the secondary grasslands in northern Europe and the Baltic countries^14^. In northern Fennoscandia, intensive forestry has expanded over the last decades, degrading much of the winter reindeer pastures but also affecting summer pastures due to e.g. intensive drainage of mires^15,16^. Land-based wind energy is also developing in the north. Wind turbines can alter grazers’ movements too, particularly in the calving season when the female reindeer avoid areas with visible turbines^17^. Other land-based industries, in particular mining, are well established in the Nordic countries and have been shown to affect reindeer husbandry^18^. Road traffic will also be higher in the vicinity of industrial facilities. Expansion of roads and other types of human infrastructure are fragmenting the landscape and reducing the accessibility of available grazing land^19^. These infrastructures pressure the herders to use trucks to move reindeer between pastures and thus force them to change their traditional practices^20^. This is already the case for sheep and cattle, where the transfer between remote pastures is generally undertaken by trucks. Tourism has also steadily increased in the Nordic countries over the last decades, with a particular interest for nature-based tourism^21^. Tourist resorts and houses are avoided by semi-domestic reindeer^22,23^. Overall, the rising human presence in northern Fennoscandia is taking place on multiple fronts, in various types of grazing land and at different spatial scales. It is therefore crucial to get a more comprehensive overview of the spatial interplay of these various land-uses in order to estimate their overall net effect on the remaining grazing land.

In addition to competing land-uses, both sheep farming and reindeer husbandry are challenged by the presence of large carnivores in Europe^24^. The management plans for Norway, Sweden and Finland aim at limiting livestock losses by restricting the abundance of these predators, in particular wolves, in the reindeer districts; by implementing monetary compensation schemes for the losses^25^; and in Norway, by setting up zones for segregating livestock (especially sheep) from predators^26^. Predator presence will affect herbivores’ behaviour and in turn change their grazing patterns by feeding in less risky habitats, but of potentially lower foraging quality^27^. If the herders are aware of the presence of a predator in the surroundings, they often move their livestock to safer grazing grounds^28^, further decreasing the potential grazing area. Altogether, the increasing pressure from concurrent land-uses results in a loss of available land for grazing.

Pressures originating from climate change are also likely to disturb the grazers and affect their grazing behaviour. For example, insect harassment would increase during warm spells in low altitude shrubby areas, making livestock more likely to stay at higher altitude^3,22^. While the presence of herbivores can appear pivotal to counteract shrub encroachment due to climate change^29–31^, avoidance of shrubby areas because of insects could trigger a negative feedback loop where shrub expansion would ultimately reduce favourable grazing areas^32^.

A comprehensive overview that summarizes the cumulative pressures from human activities and land-uses, predator presence and potential effects of climate change on northern grazing lands is currently lacking. Previous studies have addressed land-use and predator pressures at a landscape to regional scale, and only very few at a national scale. Most studies had the purpose of measuring the potential effects of the pressures on the grazers themselves without including effects of climate change^18,19,33,34^. A large-scale areal analysis of the stressors will help to delineate a spatial baseline of cumulative pressures and help diagnose the state of northern pastoralism in the context of global changes.

In this study, we estimate and map to which degree extensive domestic grazing is exposed to cumulative land-use pressures, predator presence and climate change with a main focus on their summer pastures at northern latitudes in Norway, Sweden, and Finland by using a grid-based approach advised by European standards^35,36^. With this approach, we propose a comprehensive way of analysing these pressures over large areas, enabling quantification and comparison of the extent of cumulative pressures and where these pressures co-occur with the summer grazing land. The pressures we include are outdoor tourism, land-based industrial facilities, road and railway networks, land-based wind energy, forestry, predator presence, and temperature change during the last sixty years. We specifically ask: (1) what is the overall extent of the selected pressures in northern Fennoscandia? and (2) how do these cumulative pressures overlap, and (3) how are these cumulative pressures configured over the summer grazing land? Our results hence provide a spatial baseline for projecting the expected consequences of the interplay between land-use changes, climate change and predator presence on the grazing land of northern Fennoscandia.

## Methods

### Study area

This study focuses on the northern half of Fennoscandia where reindeer husbandry and mountain farming with sheep take place^20^; hereafter referred to as “northern Fennoscandia”. We defined the extent of our study area by merging the area of all reindeer herding districts, consisting of 36% of Finland^37^, 55% of Sweden^38^ and 44% of Norway^39^. This part of Fennoscandia encompasses several bioclimatic regions, where mountain tundra and boreal forest represent the main vegetation zones^40^.

### Study system

Our study area has a long history of reindeer herding, where practices vary between regions. A total of 188 reindeer herding districts, spread over northern Fennoscandia, are exclusively run by Sámi people in Sweden and in most of Norway, whereas both Sámi and ethnic Finns are herders in Finland^2^. In Norway and a major part of Sweden, most districts follow a migratory pattern between the coast and the mountains, but in Finland and some districts in Sweden, there is also year-round grazing^41^. In most districts in Sweden, migrating reindeer tend to spend the summer grazing in the mountain tundra and winter in the forests east of the mountains^41^. In Norway, migrating districts show the opposite pattern with summer grazing along the western coast and winter grazing in the mountains towards the Swedish, Finnish or Russian borders^42^. Year-round herding districts in Sweden and Finland stay in the boreal forest all year. Mountain farming with livestock other than reindeer and based on transhumance also takes place in northern Fennoscandia, but it has decreased dramatically since the nineteenth century^43^. In Norway, where sheep husbandry is most developed (up to 15,000 herds)^44^, grazing is usually organised in grazing units (Norw: beitebruk), where farmers can gather their livestock in communal extensive pastures^45^. Fewer farmers, c. 200, are practicing transhumance (Sw: fäbodbruk) in Sweden^46^, and c. 150 goat and sheep farmers are active in northern Finland^47^.

### Spatial overview of the grazing areas

We collected land-cover data from Corine Land Cover 2018, (spatial resolution of 100 × 100 m)^48^ for our study area to contrast the quality of the summer grazing land. Each land-cover category was labelled into a potential degree of foraging value by the domestic and semi-domestic grazers, based on the definitions of the land-cover classes^49^, expert knowledge, and previous studies on habitat preferences of sheep and reindeer in summer^22,32,50^. This reclassification of the land-cover resulted in a “potential grazing value” made of three levels: high, medium, and low value (Table 1).

**Table 1:** Reclassification table of the Corine land-cover types in degree of usage by the grazers in summer according to expert knowledge and literature^22,32,50^.

Corine land-cover code	Land-cover type in Northern Fennoscandia	Potential grazing value
231	Pastures	High
243	Land principally occupied by agriculture, with significant areas of natural vegetation	High
311	Broad-leaved forest	High
321, 322, 324	Natural grasslands, moors and heathland, transitional woodland-shrub	High
412	Peat bogs	High
242	Complex cultivation patterns	Medium
333, 334	Sparsely vegetated areas, burnt areas	Medium
313	Mixed forest	Medium
411	Inland marshes	Medium
312	Coniferous forest	Low
421	Salt marshes	Low
111, 112, 121, 122, 123, 124, 131, 132, 133, 141, 142	Artificial surfaces	No grazing (N/A)
211	Arable land	No grazing (N/A)
222	Permanent crops	No grazing (N/A)
331, 332, 335	Beaches, dunes, sands, bare rocks, glaciers and perpetual snow	No grazing (N/A)
423, 511, 512, 521, 522, 523	Intertidal flats and water bodies	No grazing (N/A)

### Review of the cumulative pressures

Spatial data on land-use, predator presence, and climate was collected for the study area. Details about data collection can be found in supplementary material (Table S1). Five types of land-use were mapped: outdoor tourism, land-based industrial facilities, road and railway networks, land-based wind energy, and forestry. Since outdoor tourism is widespread in Scandinavia, locations of mountain stations, huts, and mountain hostels, and their respective bed capacities, were collected by parsing through national outdoor tourism websites for the three countries (Table S1). Locations of private cabins (such as summer huts, holiday homes, or secondary residences) were also downloaded from national land survey platforms (Table S1). Locations of active mines were downloaded from the Fennoscandian Mineral Deposits website and locations of land-based industrial facilities registered on the European Inspire platform for sharing open data on pollution were also collected^51^. These facilities encompass various kinds of industrial activities that are registered for having a permit for environmentally hazardous activities and are listed on the public authorities’ web platforms (Table S1). Regarding land-based wind energy, we collected the locations of the wind farms in use and planned to be built, as well as their respective number of wind turbines on the national web platforms (Table S1). The railway network and the main roads (typically wider than 5 m) consisting of European, national and county roads in use were collected from public authorities’ web platforms (Table S1). The forestry map was derived from a likelihood model of forest management regime developed by Schulze et al.^52^, made of different classes, and from which we included the type of forest classified as primarily used for production. Range maps of presence of large mammal predators (bear *Ursus arctos*, lynx *Lynx lynx*, wolf *Canis lupus,* and wolverine *Gulo gulo*) were obtained from Kaczensky et al.^25^ for the period of 2015–2017. Golden eagle (*Aquila chrysaetos*) is another predator of sheep and reindeer in Fennoscandia, but a presence map for this species could not be accessed for the three countries, and was therefore not included in this study. To observe changes in climate, and not only weather, data from 30-year periods or more are commonly used^53^. Here we selected the two last 30-year periods to detect changes with time. Climate trends were drawn by collecting historical and current monthly surface temperature and precipitation (from 1959 to 2018) over northern Fennoscandia from models developed by the Climate Research Unit (CRU)^54^.

### Geographic information system (GIS) and statistical analysis

Statistical analysis and data-handling of the different GIS layers were performed in R^55^ (with the packages ‘raster’, ‘rgdal’, ‘rgeos’, ‘ncdf4, ‘gdalUtils’, ‘car’, ‘mblm’, ‘mapview’ and ‘spatialEco’), and QGIS (version 3.4.7, accessed on www.qgis.org). Details about data handling and statistical analysis in R can be found in the supplementary material (‘source code’ S2). Once collected, the different data layers were cleaned by selecting only land-uses and infrastructures in use, based on available metadata. Duplicates were excluded. Each of the studied pressures had a distinct areal impact which was not possible to map in absolute terms at this scale, which is why we used a grid-based approach advised by European guidelines^35,36^. Hence, to make the collected datasets uniform in terms of accuracy and resolution, the layers were framed over a grid of 10 km × 10 km cell (European reference grid)^56^. The sum of bed capacity of the outdoor tourism accommodations and the sum of private cabins were calculated for every grid cell. Norway and Finland had similar ways of categorising private cabins, but not Sweden which may result in a slightly underestimated accuracy compared to the two other countries. Main road and railway density was computed (in km/100 km^2^) for every grid cell. Since we could not access the extent of the land-based industrial facilities, their presence/absence was extracted from the grid. The total sum of wind turbines was computed for every grid cell. Predator pressure was estimated by only including the permanent presence of predators, and excluding their sporadic presence. This pressure was then calculated by counting the number of predator species permanently present in each grid cell.

Climatic trends were drawn by performing linear and quadratic regressions over 60 years (1959 to 2018) for all the grid cells in the study area. No trends for precipitation change were found and precipitation was therefore excluded from the analysis. As for temperature change, if the quadratic models were a better fit than the linear models, the quadratic models were then used to calculate temperature change over time by selecting the slope for the year in the middle of the 60 years period (1988). For all models, autocorrelation and normality of the residuals were checked.

The extent of the grazing areas and of the multiple disturbances were then mapped and analysed according to the potential grazing values (Table 1). To map and investigate the co-occurrence of the pressures, we estimated an index of the cumulative pressures co-existing for each grid cell over the full study area. The extent of the different land-uses and the predator species presence were then added up into one map. For this cumulative index, forestry was accounted for if the grid cells contained more than 50% forests primarily used for wood production. The road and railway density were accounted for with a minimum of 1 km per grid cell. The extent of outdoor tourism was accounted for if at least an outdoor tourism accommodation or a private cabin was present in a grid cell. These thresholds may appear conservative, but single human infrastructures can cause grazers to avoid the area by up to 15 kms away^34^, and hence create an area of disturbance around it^19,34,57^. The size of this area of disturbance is context-dependent and will change depending on the type of construction^34^. The cumulative pressures were then calculated where a grid cell could contain a value between zero (no pressures) up to nine (five different land-uses and four predator species). The main objective with this map was not to analyse the effects of these pressures, separately or together, but rather to map where they take place concurrently. The maps were produced in QGIS (version 3.4.7, accessed on www.qgis.org).

## Results

### Spatial overview of the grazing areas

**Figure 1:**
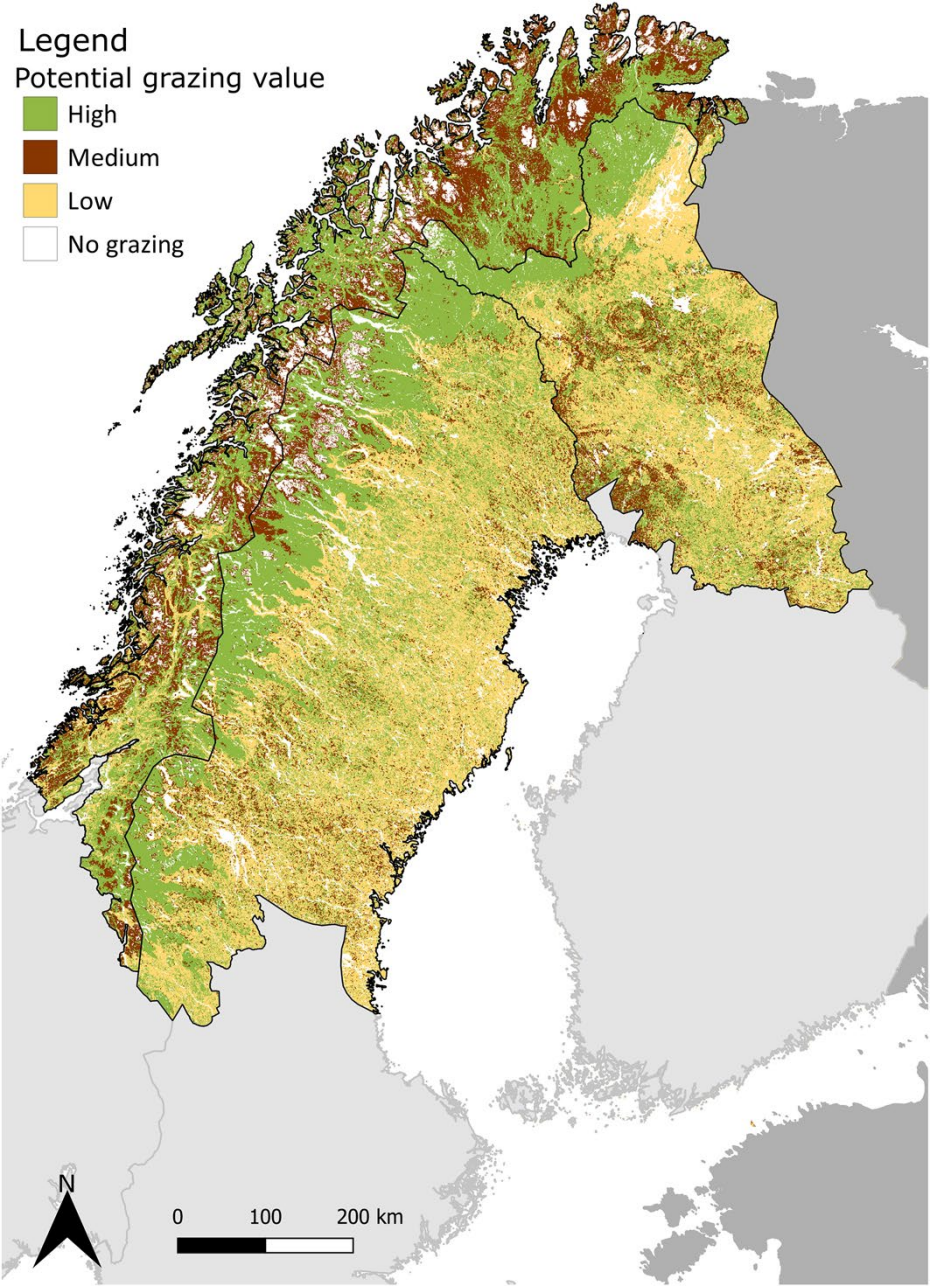
Map of the potential grazing values in summer pastures of northern Fennoscandia. The reclassification is based on Corine land-cover 2018 (detailed in Table 1). This map was created with QGIS version 3.4.7, accessed on www.qgis.org. The country borders were from Eurostat ©EuroGeographics.

We reclassified the land-cover of northern Fennoscandia into potential grazing values to pinpoint the summer pastures (Table 1). Using this reclassification, the region was made up of 38% potentially high grazing value (195,672 km^2^), 17% potentially medium grazing value (86,275 km^2^), 34% potentially low grazing value (176,088 km^2^), and 11% without grazing value (54,137 km^2^) (Fig. 1).

### Review of the cumulative pressures

**Figure 2:**
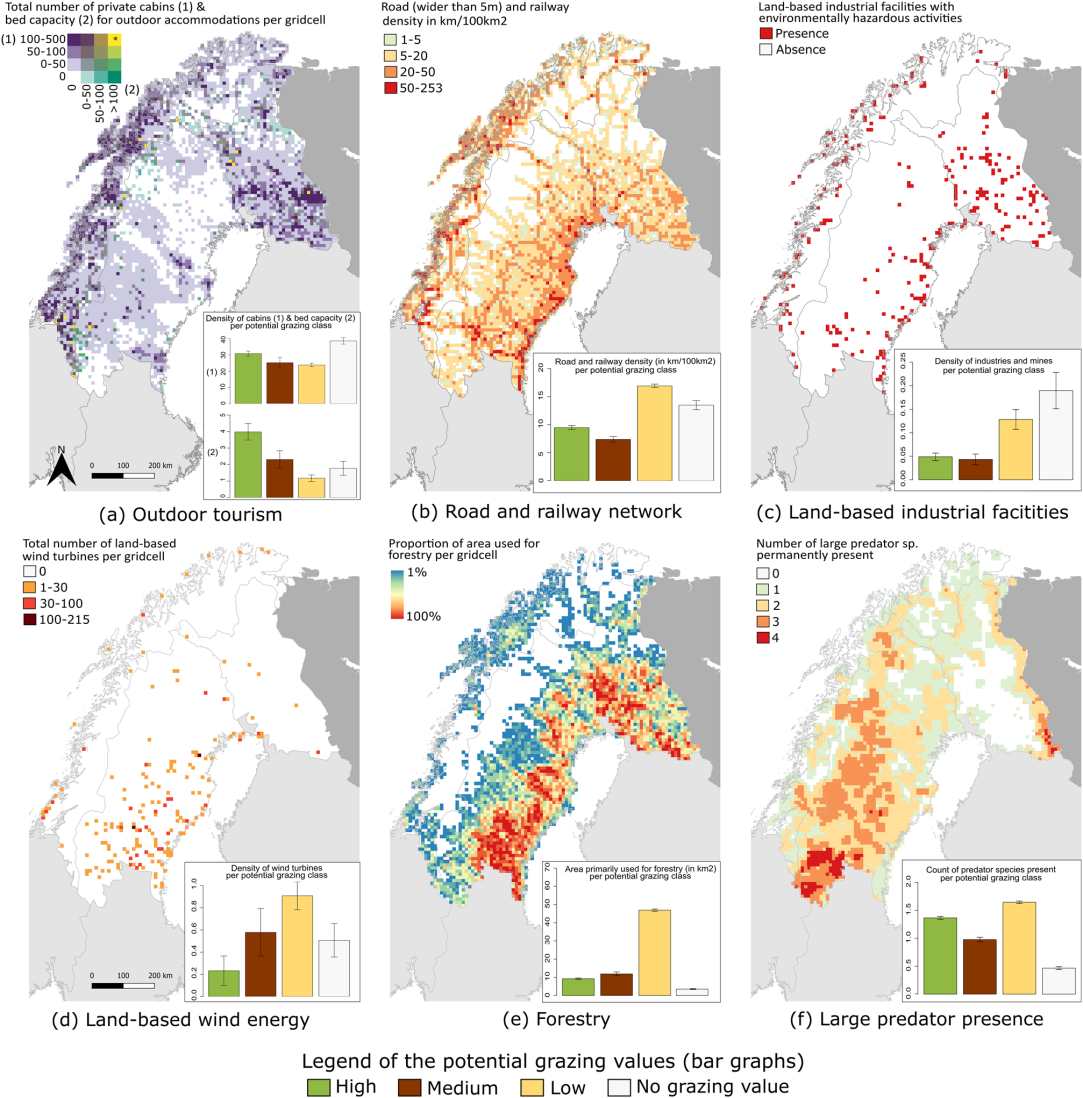
Maps over northern Fennoscandia depicting the (**a**) private cabins and outdoor tourism accommodations, (**b**) road and railway network, (**c**) land-based industrial facilities, (**d**) land-based wind energy, (**e**) the proportion of area primarily used for forestry, (**f**) the number of large predator species permanently present. For each map, the distribution of the pressure per potential grazing value is given with bar graphs. The error bars show standard errors. These maps were created with QGIS version 3.4.7, accessed on www.qgis.org. The country borders were from Eurostat ©EuroGeographics. *Note that, for Fig. 3a, for eight of the yellow grid cells, the total number of private cabins exceeds 500 and don’t contain any tourism accommodations.

We found a total of 595 outdoor tourism accommodations with an average capacity of 21.3 ± 1.3 beds, that covered 8% of northern Fennoscandia. Most of them were located in areas with potentially high and medium grazing quality, with an average bed capacity of 3.98 ± 0.5 and of 2.31 ± 0.52 respectively (Fig. 2a). A total of 162,021 private cabins were spread over 69% of the study region primarily in areas without grazing value (average density of cabins of 38.68 ± 2), but were also present extensively in areas with high, medium and low grazing quality (average density of 30.92 ± 1.53, 25.26 ± 3.26, 23.83 ± 1 respectively). Overall, when accounting for the extent of outdoor tourism accommodations and private cabins, outdoor tourism covered 71% of northern Fennoscandia. The road and railway network extended over 66% of the region, mainly in areas of potentially low grazing value (density of 16.9 ± 0.32 km/100 km^2^) and without grazing value (density of 13.46 ± 0.8 km/100 km^2^, Fig. 2b). 600 industrial facilities were distributed across 5% of northern Fennoscandia, mostly concentrated to areas without or with low grazing value (density of 0.18 ± 0.03 and of 0.12 ± 0.02 respectively, Fig. 2c). Further, 235 wind farms with a mean number of 14.59 ± 1.41 wind turbines covered 3% of the region (with a total of 3428 wind turbines), essentially in the areas with potentially low grazing value (with an average wind turbine density that was of 0.9 ± 0.12, Fig. 2d). Forests managed for wood production covered 67% of northern Fennoscandia (141,933 km^2^), also mostly present in the areas of potentially low summer grazing value (Fig. 2e). The four large predators (bear, lynx, wolf and wolverine) were permanently present, with at least one predator species over 71% of the region (Fig. 2f). In areas with potentially low, medium, and high grazing value respectively, there was an average of 1.64 ± 0.02, 0.97 ± 0.04 and 1.36 ± 0.02 predator species, while in the non-grazing areas, this average was of 0.46 ± 0.02.

**Figure 3:**
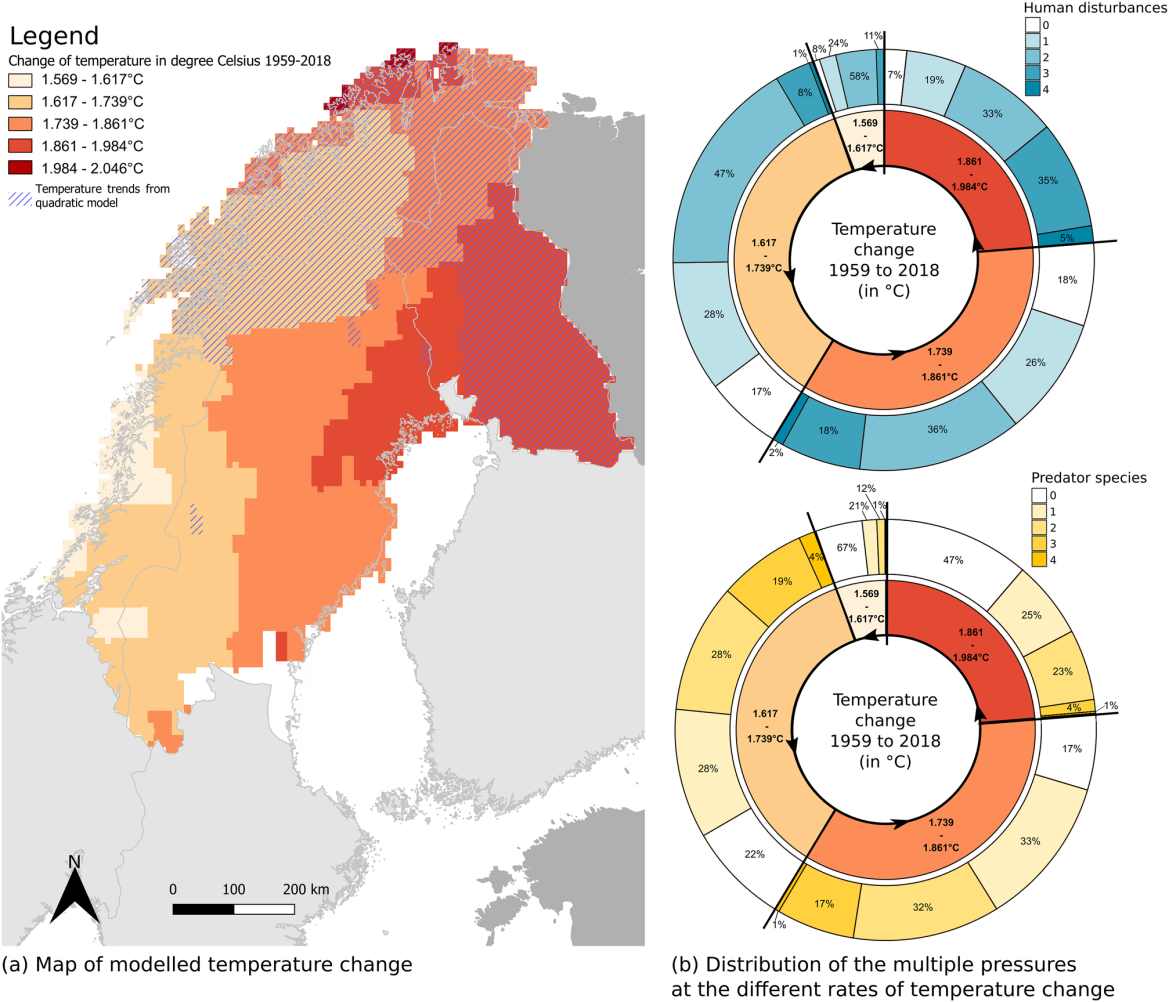
(**a**) Temperature changes over northern Fennoscandia for the last 60 years (1959 to 2018) coloured in shades of red (see legend). The residuals of all the models (linear and quadratic) were normal. The area covered by blue hatched lines are the areas where the quadratic models were a better fit than the linear models, and were therefore used to model temperature change over time. The residuals of these models were normal and not auto-correlated. 4.3% of the linear models were serially auto-correlated, therefore excluded and shown in white. This map was created with QGIS version 3.4.7, accessed on www.qgis.org. The country borders were from Eurostat ©EuroGeographics. (**b**) Distribution of the multiple pressures (co-occurring land-uses in shades of blue and co-occurring predator species in shades of yellow) at the different rates of temperature change up to 1.984 °C. For each specific rate of temperature change, the percentages show the extent (in number of grid cells) under cumulative pressures. Note that six grid cells contained five land-uses but were not included in this graph for visual purposes.

We analysed the temperature trends for every grid cell in the area. For all grid cells, there was a significant increase in temperature over the last 60 years (1959–2018) ranging from 1.5 °C in the south-west to 2 °C in the north-east of northern Fennoscandia (Fig. 3a), with more than 90% of the grid cells ranging from 1.6 to 1.9 °C (Fig. 3b). A marginal area with the strongest temperature changes (between 1.98 and 2.05 °C) was on the north coast of Norway (0.4% of the grid cells, Fig. 3a and excluded in Fig. 3b for visual purposes). 45% of the grid cells showed a better fit with a quadratic model than with a linear model (blue hatched lines in Fig. 3a), implying that the rate of temperature change was not constant over the 60 years period, due to stronger increase in temperatures in recent years. These large temperature changes over time were observed over northern Norway, the tip of northern Sweden, and the eastern part of northern Finland. In areas with a high rate of climate warming (up to 1.98 °C), we could also observe a high number of co-occurring land-uses (Fig. 3b).

**Figure 4:**
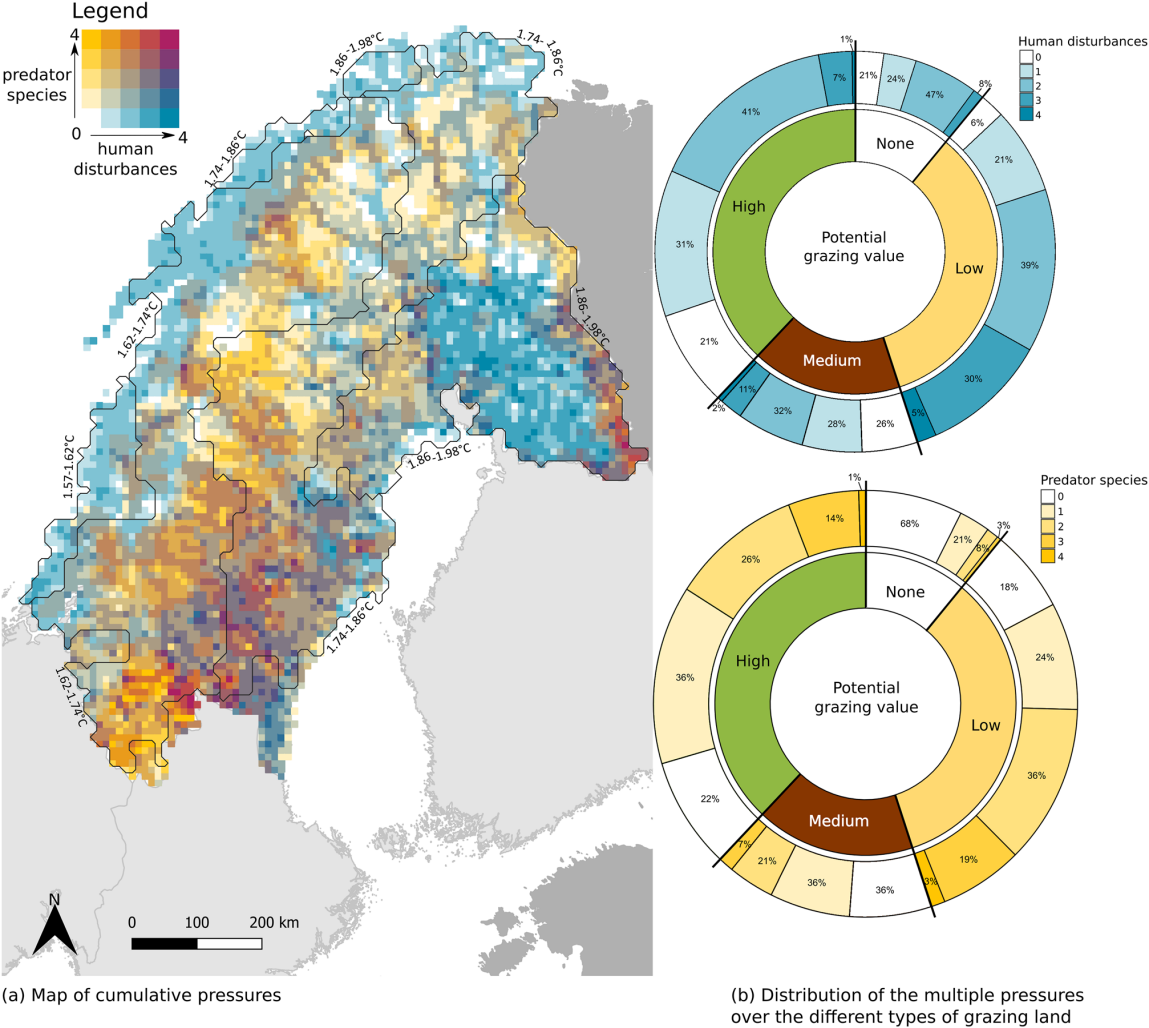
(**a**) Map showing the cumulative pressures affecting extensive domestic grazing over northern Fennoscandia. Temperature changes modelled for 1959 until 2018 are depicted with contour lines. Note that the bivariate colour legend ends at four land-uses co-occurring in one grid cell for visual purposes, but six grid cells contained five land-use pressures, as well as predator presence (five grid cells were located in central Sweden and one in Finland next to the Russian border). This map was created with QGIS version 3.4.7, accessed on www.qgis.org. The country borders were from Eurostat ©EuroGeographics. (**b**) Distribution of the multiple pressures (co-occurring land-uses in shades of blue and co-occurring predator species in shades of yellow) over the different types of grazing land. For each potential grazing value, the percentages show the extent (in number of grid cells) under cumulative pressures. Note that six grid cells contained five land-uses but were not included in this graph for visual purposes.

When compiling the human land-uses into one map, we found that 85% of the study area was covered by at least one land-use pressure, and that 60% of the area contained several land-use pressures (two or more, Fig. 4a). Multiple land-uses (two or more, Fig. 4b) was mostly present in grazing land with low grazing value (74%), but also in almost half of the grazing land with potentially high (49%) and medium grazing values (45%). Human land-uses were concentrated into a stretch reaching from central-eastern Sweden up to central Finland and present all along the coast of Norway. The Fennoscandian mountain range was intersected by the road and railway network and the presence of outdoor tourism, but also dominated by predator presence (Figs. 2 and 4a). A continuous predator presence also followed the Russian border in Finland, indicating a region with cumulative pressures in central-eastern Finland, with four predator species permanently present and three to four land-use activities occurring at the same place (Fig. 4a). The majority of all grazing land (high, medium and low grazing values) coincided with the permanent presence of at least one predator species (Fig. 4b). Undisturbed areas without any permanent presence of predators, nor co-occurring land-uses, were small and scattered throughout the Scandinavian mountain range up to the northern tip of Norway and to a small patch in north-east Finland, only representing 4% of the whole study area. More than half of northern Fennoscandia (60%) was occupied by at least one human disturbance and by at least one predator species. No region with four predator species and five land-uses was found, but areas made of eight cumulative pressures were located in central Sweden, not far from the southern extent of the study area. Areas with seven cumulative pressures were found in Norway, central Sweden, and next to the Russian border in Finland (Fig. 4a). Both the north coast of Norway, and most of northern Finland, were predominantly affected by human disturbances (Fig. 4a).

## Discussion

This study delineates for the first time a spatial baseline of the various land-use, climate, and predator pressures affecting free-ranging domestic and semi-domestic grazing in the northern parts of Sweden, Norway and Finland. With our extensive grid-based approach, a major finding was that although 40% of Fennoscandia has been assigned as pastures for traditional herding^3,4^, only 15% is left undisturbed from the competing human land-uses that we have included in this study. Note that this overview remains rather conservative as we have not been able to include all infrastructures that can disturb grazers—e.g. hydroelectric dams or power lines.

Only around a third of this region is made of potential high grazing value for summer grazing. This high-quality summer grazing land is concentrated over the Fennoscandian mountain range, i.e. summer pastures used primarily by migratory reindeer, while half of it coincides with several human disturbances. Our study shows that the different pressures were indeed present in all types of grazing land, but not equally distributed. While outdoor tourism occurs largely in areas of potential high grazing value, the other land-uses (road and railway network, land-based industries and wind energy, as well as forestry) co-occur mostly in the potentially low grazing value areas. This pattern could indicate an expansion of outdoor tourism at the heart of the summer grazing land. In past decades, Norway has faced unprecedented challenges due to an increasing interest for nature-based tourism^21^. Similarly, yet to a lesser extent, summer guest night statistics in the mountain stations of northern Sweden have increased of 30% since 1988^58^, reaching new records since 2019^59^. Since tourism in mountain areas is increasing, some studies are proposing to channel the tourists to specific infrastructures at the entrance of protected areas away from the sensitive areas^21,60^.

In our study region, forestry is the second-most widespread land-use, covering 67% of northern Fennoscandia. Forestry occurs mostly in coniferous forests that are of low value for summer grazing but high value for winter grazing of reindeer. This large extent reveals a strong pressure on free-ranging grazing, in particular for the non-migratory reindeer districts, as well as for the winter pastures^15^. Compared to most other land-use pressures, forestry covers large areas and is a highly dynamic land-use with varying degrees of intensity. This is different from the less human-intrusive land-based wind energy, which occupies 3% of the region and co-occur with forestry, as well as with the road and railway network. We show that 5-m wide roads and railways are also present over a large part of northern Fennoscandia (66%). As they are known to be avoided especially by reindeer by 1 to 1.5 km^23,34^, these structures have a fragmenting effect on the landscape and disrupt migratory routes^61^. Land-based industrial infrastructures cover 5% of northern Fennoscandia and occur mostly in areas where grazing cannot take place. Although these activities occupy a relatively small area, they reflect a growing land-cover conversion caused by the implementation of these activities, reducing pasture accessibility, disturbing migratory routes, and hence a general loss of grazing land^57^. Since these competing land-uses take place over 85% of northern Fennoscandia, this has affected the economy of animal husbandry^62^, and could cause a decline in livestock numbers, especially for reindeer. Yet, the semi-domesticated reindeer populations in Norway and Finland were still recovering from the strong decline happening during the Second World War up until the 2010s^62^. Since then, semi-domestic reindeer numbers remained fairly constant for the three Nordic countries, which could be partly explained by the recent use of supplementary feeding in winter^62^. Ultimately, access to food in winter in essential for reindeer survival^3^, and while our study focuses on the summer grazing areas, our maps could also be of relevance to study cumulative pressures in regard to winter pasture use.

In addition, the presence of large predators also adds a top-down pressure on the livestock. Four large predators (lynx, wolverine, bear and wolf) are permanently present in northern Fennoscandia, with at least one predator species present over 71% of the area. Despite strict governmental management plans regarding predator presence in the reindeer herding districts^25^, our results show four regions where all four predators are present, indicating a strong pressure on free-ranging livestock. This result should however be interpreted with caution because interactions between predator species could affect the overall predation pressure on grazers differently depending on species and abundance^63^. For example, it is suspected that the presence of lynx can indirectly reduce the predation rate of wolverine on reindeer due to more scavenging opportunities^64^. Our cumulative amount of species should therefore be used as an index of disturbance towards grazing patterns rather than an index of killing rate. Moreover, the assumption that the co-occurrence of the several pressures always results in cumulative negative effects is probably an over simplification^65^ and our mapping of pressures should therefore be used carefully, especially regarding the interactions between predators and land-use pressures, but also with climate change. These interactions could trigger unknown outcomes that could potentially indirectly benefit the grazers. For example, if large predators avoid high human activity areas, it could create a refuge from predation in those areas^66^. Yet, to our knowledge, such pattern has not yet been reported with semi-domestic reindeer. However, if unknown synergies remain between the different pressures, we still assume that the co-occurrence of these pressures on the grazing land will weaken the flexibility that northern pastoralism needs to survive. In the case of predators, their presence will disperse the grazers, disturb pasture use, and limit access to high quality grazing areas^27,41^, which will require more flexible use of the grazing land^67^ but is then limited by other human activities. This is the case for almost half of northern Fennoscandia, where the area was occupied by at least one human disturbance and by at least one predator species (Fig. 4a). If livestock are moved from areas with high predation risk^26,28^ and at the same time avoid areas with anthropogenic disturbances^34^, this would inevitably lead to intensified grazing in some areas, and abandonment of others.

Climate change is yet another significant pressure on ecosystems at high latitudes^8^. Our models indicate an increase in temperature in the region from 1.5 to 2 °C over the last 60 years. In the long run, such a temperature rise could trigger biome shifts, with increasing primary productivity and expansion of boreal species northwards^9–11^. Reindeer husbandry is often seen as a means for preserving the mountain landscape according to the Swedish and Norwegian environmental objectives, and free-ranging grazing may counteract climate-driven changes on vegetation, but this would be without considering the interplay with the co-existing cumulative pressures. Overall, the persistence of reindeer herding in a changing climate relies on the capacity of the herding system to adapt to unpredictable weather conditions^3^, but this adaptation capacity is now diminished by the expansion of competing land-uses and predator pressure^3,41,68^. According to our results, human activities are dominating the study region, exposing grazers to cumulative pressures and potentially constraining their grazing area. We stress that climate-driven changes on plant communities are likely to have a synergetic or additive effect in regions with many cumulative pressures (located in Norway, central-eastern Finland and central Sweden), and these effects may occur faster as livestock grazing is constrained. The synergetic effects of a changing climate and intensified land-use risk causing an increase of fragmented pastures leading to more concentrated grazing pressures that could negatively affect plant communities^32,69^. High levels of grazing pressure from sheep and reindeer is hence of high concern because it could compromise the quality and the functioning of the grazing system^70,71^. Therefore, it is not only the amount of grazing land that is important to monitor, but also its configuration in the landscape in order to fully understand what types of disturbances the grazers are exposed to, e.g. proximity of human settlements, industries, roads and predators.

Addressing the co-occurrence of cumulative pressures remains a complex task since the pressures’ impacts are context-dependent and hard to foresee^57^. With our grid-based approach, the models are static, coarse and hence somewhat simplistic to be able to produce an overview at this spatially extensive scale. This technique, however, enabled us to be free from the local context and to compare the spatial distribution of pressures that act at very different scales. Our overview indicates that very different land-uses co-occur in many areas of northern Fennoscandia, but also together with predator species that are permanently present, in a changing climate. This highlights the need for more interdisciplinary research that focuses on the interactions between land-uses, species interactions and global warming. This study also shows a high proportion of area under cumulative impacts, with 60% of northern Fennoscandia containing at least two co-existing land-use pressures. Considering the large expansion of human activities over this grazing land during the last decades^33,57^, we see no reason why these cumulative effects would not expand in the future. This confirms that northern pastoralism across 40% of Fennoscandia is exposed to increasing pressures from concurrent land-uses as well as large predators and climate change, thereby demonstrating the importance for land-use and climate change to be mapped and analysed together to correctly inform management policies. We hope our results will act as a first step towards a better comprehension of global changes in northern ecosystems and can serve as guidance on how to map and analyse traditional grazing areas under cumulative impacts.

## Supplementary Information

Supplementary Table S1.

Supplementary Information 2.

## Data Availability

All the data used in our analyses are available online, with their URL links available in the Supplementary Information S1. The final compiled dataset, as well as the source code used for statistical analysis and compilation, is made available as Supplementary Zip File S2.
