# Supplementary material for: Mapping cumulative pressures on the grazing lands of northern Fennoscandia
Source: Sci Rep. 2022 Sep 30;12:16044. doi: 10.1038/s41598-022-20095-w (PMC9525264; doi:10.1038/s41598-022-20095-w)
Supplement: Supplementary file 1 — Supplementary Table S1. [file 41598_2022_20095_MOESM1_ESM.pdf]

Mapping cumulative pressures on the grazing lands of northern Fennoscandia

Marianne Stoessel*[1,2], Jon Moen[3], and Regina Lindborg[1,2]

[1]: Department of Physical Geography, Stockholm University, SE-106 91 Stockholm, Sweden

[2]: The Bolin Centre for Climate Research, Stockholm University, SE-106 91 Stockholm, Sweden

[3]: Department of Ecology and Environmental Science, Umeå University, SE-901 87 Umeå, Sweden

*marianne.stoessel@natgeo.su.se ; https://orcid.org/0000-0001-6976-8139

Supplementary material, Table S1: List of the data collected, together with the data provider, spatial resolution, latest access date and URL link

Data	Provider	Spatial data type & resolution	Latest access date	URL
CORINE Land Cover	Copernicus land monitoring service	Raster (100x100m)	27 Feb 2019	https://land.copernicus.eu/pan-european/corine-land-cover/clc2018
Forest management regimes	Schulze et al. 2019	Raster (1x1km)	26 Feb 2019	https://www.environmentalgeography.nl/site/data-models/data/forest-classes-and-uses/
Outdoor tourism accommodations in Sweden	The Swedish Tourist Association STF	Point Shapefile	20 Feb 2019	https://book.stfturist.se/en/accommodation?filter=c=22409 the GPS locations were extracted via ParseHub
Outdoor tourism accommodations in Norway	The Norwegian Trekking Association UT	Point Shapefile	27 Nov 2018	https://ut.no/utforsker/norge/hytter the GPS locations were extracted via ParseHub
Outdoor tourism accommodations in Finland	The official website of parks and wildlife Finland	Point Shapefile	16 April 2019	https://www.utinaturen.fi/stugor/ the GPS locations were extracted via ParseHub
Private cabins in Sweden	The Swedish Land Survey	Polygon shapefile	4 June 2021	https://zeus.slu.se/get/
Private cabins in Norway	The Norwegian Mapping Authority	Point shapefile	26 May 2021	https://register.geonorge.no/register/versjoner/produktspesifikasjoner/kartverket/matrikkelen-bygningspunkt
Private cabins in Finland	The National Land Survey of Finland	Polygon shapefile	7 June 2021	https://www.maanmittauslaitos.fi/en/maps-and-spatial-data/expert-users/topographic-data-and-how-acquire-it
Road and railway network In Sweden	The Swedish Transport Administration	Line Shapefile	24 April 2019	https://lastkajen.trafikverket.se/
Road network In Norway	The Norwegian Public Roads Administration	Line Shapefile	8 Jan 2021	https://www.vegvesen.no/vegkart/vegkart/#kartlag:geodata/hva:(~(id:777,filter:(~),farge:'0_0))/@600000,7225000,3
Railway network in Norway	The Norwegian national railway infrastructure	Line Shapefile	25 March 2019	https://kartkatalog.geonorge.no/metadata/uuid/c3da3591-cded-4584-a4b1-bc61b7d1f4f2
Road and railway network in Finland	The Finnish transport agency	Line Shapefile	13 Dec 2018	https://julkinen.liikennevirasto.fi/oskari/
Wind turbines in Sweden	The Swedish Energy Agency	Point Shapefile	5 March 2019	https://vbk.lansstyrelsen.se/sv
Wind turbines in Norway	The Norwegian Water Resources and Energy Directorate	Point Shapefile	14 Dec 2018	http://nedlasting.nve.no/gis/
Wind turbines in Finland	The Finnish Wind Power Association & Etha Wind	Point Shapefile	26 Feb 2019	https://www.ethawind.com/suomen-tuulivoimapuistot/ and https://www.tuulivoimayhdistys.fi/hankelista
Active Mines of Fennoscandia	The Fennoscandian Mineral Deposits	Point Shapefile	28 Jan 2019	https://hakku.gtk.fi/en/locations/search?location_id=109
Land-based industries In Norway	The Norwegian Environmental Agency	Point Shapefile	28 Feb 2019	https://kartkatalog.miljodirektoratet.no/Dataset/Details/60
Land-based industries In Sweden	The Swedish county administrative board	Point Shapefile	28 Feb 2019	https://ext-geodatakatalog.lansstyrelsen.se/GeodataKatalogen/
Land-based industries In Finland	Statistics Finland	Point Shapefile	27 March 2019	http://www.stat.fi/org/avoindata/paikkatietoaineistot.html
Large predator's permanent range	Kaczensky et al., 2018 (updated from 2013)	Grid Shapefile (10x10km)	22 Nov 2019	https://ec.europa.eu/environment/nature/conservation/species/carnivores/conservation_status.htm
Monthly surface air temperature and monthly precipitation, 1901 to 2018	Climate Research Unit (Harris et al. 2014)	NetCDfiles (0.5°x0.5°)	30 April 2019	https://crudata.uea.ac.uk/cru/data/hrg/
